# Reply regarding: A 3-mm protrusion threshold after pediatric forearm ESIN: a rule-out safety margin, not a decision rule

**DOI:** 10.2340/17453674.2026.46258

**Published:** 2026-06-12

**Authors:** Jan Egil BRATTGJERD, Christer AASHEIM, Astrid ROSENBERG, Christoffer FOTLAND, Vera HALVORSEN

**Affiliations:** 1 Division of Orthopaedic Surgery, Oslo University Hospital, Oslo;; 2 Institute of Clinical Medicine, Faculty of Medicine, University of Oslo, Oslo;; 3 Institute of Rehabilitation Science and Health Technology, Faculty of Health Science, Oslo Metropolitan University, Oslo, Norway

*Sir,–*We thank Zhang and Lu for their comments and agree that the 3.3-mm threshold is best interpreted as a rule-out safety margin rather than a decision rule [1]. This was also our intended interpretation, as implant removal should remain symptom-driven, whereas the threshold may guide nail impaction when implant retention is pursued [2].

We appreciate their use of the “rule of three,” which supports our main finding that protrusion below 3 mm was associated with a very low risk of irritation-related implant removal.

Later extraction is an important consideration when implant retention is intended. We agree that deeper impaction may increase extraction difficulty. However, extraction-related events were uncommon in our cohort. The number of such events was too small to determine whether impaction depth influenced later extraction. Notably, all irritation-related extractions occurred in nails with protrusion above the threshold.

We also agree that protrusion is unlikely to be the sole determinant of local symptoms. Delayed discomfort occurred in some patients without measurable protrusion. This suggests that factors other than nail protrusion may contribute to symptom development.

Further studies are needed. Larger cohorts may allow multivariable analyses of factors associated with local symptoms and implant retention, and prospective studies may allow standardized assessment of symptoms and extraction difficulty. While comparative studies are needed to define the role of nail impaction relative to conventional ESIN management, our study evaluated outcomes within a retention strategy. Nevertheless, the main clinical message remains unchanged. If implant retention is intended, nail protrusion should be minimized.


**Jan Egil BRATTGJERD ^1–3^, Christer AASHEIM ^1,2^, Astrid ROSENBERG ^1,2^, Christoffer FOTLAND ^1^, and Vera HALVORSEN ^1^** *^1^ Division of Orthopaedic Surgery, Oslo University Hospital, Oslo;* *^2^ Institute of Clinical Medicine, Faculty of Medicine, University of Oslo, Oslo;* *^3^ Institute of Rehabilitation Science and Health Technology, Faculty of Health Science, Oslo Metropolitan University, Oslo, Norway* *Correspondence: jbratt@ous-hf.no*


## Notes

Complete disclosure of interest forms according to ICMJE are available on the article page, doi: 10.2340/17453674.2026.46258
